# Vitamin D, oral health, and disease characteristics in juvenile idiopathic arthritis: a multicenter cross-sectional study

**DOI:** 10.1186/s12903-022-02349-1

**Published:** 2022-08-08

**Authors:** Lena Cetrelli, Athanasia Bletsa, Anette Lundestad, Elisabet Grut Gil, Johannes Fischer, Josefine Halbig, Paula Frid, Oskar Angenete, Ingrid Lillevoll, Annika Rosén, Karin B. Tylleskär, Keio Luukko, Ellen Nordal, Anne Nordrehaug Åstrøm, Marit Slåttelid Skeie, Astrid Jullumstrø Feuerherm, Abhijit Sen, Marite Rygg

**Affiliations:** 1 Center of Oral Health Services and Research (TkMidt), Trondheim, Norway; 2 The Public Dental Health Service, Trøndelag County, Trondheim, Norway; 3 Department of Clinical and Molecular Medicine, Faculty of Medicine and Health Sciences, Norwegian University of Science and Technology (NTNU), Trondheim, Norway; 4 Oral Health Centre of Expertise in Western Norway (TkV), Bergen, Norway; 5 Department of Clinical Dentistry, The Faculty of Medicine, University of Bergen, Bergen, Norway; 6 Department of Pediatrics, St. Olavs University Hospital, Trondheim, Norway; 7 Public Dental Health Service Competence Centre of Northern Norway (TkNN), Tromsø, Norway; 8 Department of Clinical Medicine, The Arctic University of Norway, Tromsø (UiT), Norway; 9 Department of Clinical Dentistry, The Arctic University of Norway, Tromsø (UiT), Norway; 10 Division of Oral and Maxillofacial Surgery, Department of Otorhinolaryngology, University Hospital North Norway, Tromsø, Norway; 11 Department of Radiology and Nuclear Medicine, St Olav Hospital HF, Trondheim University Hospital, Trondheim, Norway; 12 Department of Circulation and Medical Imaging, Faculty of Medicine and Health Sciences, Norwegian University of Science and Technology, Trondheim, Norway; 13 Department of Oral and Maxillofacial Surgery, Haukeland University Hospital, Bergen, Norway; 14 The Children’s Clinic at Haukeland University Hospital, Bergen, Norway; 15 Department of Pediatrics, University Hospital of Northern Norway, Tromsø, Norway

**Keywords:** Rheumatic disease, Juvenile arthritis, Disease activity, Children, Adolescents, 25(OH) vitamin D, Vitamin D insufficiency, Oral health, Dental caries, Gingival inflammation

## Abstract

**Background:**

Vitamin D deficiency has been associated with autoimmune diseases and oral health. Knowledge about the association between vitamin D status and oral conditions in JIA is limited. We aimed to investigate vitamin D status in a cohort of Norwegian children and adolescents with JIA and possible associations between serum vitamin D levels, clinical indicators of oral health, and JIA disease characteristics.

**Methods:**

This multi-center, cross-sectional study, included individuals with JIA aged 4–16 years from three geographically spread regions in Norway. Demographic data, age at disease onset, disease duration, JIA category, disease status, medication, and vitamin D intake were registered. One blood sample per individual was analyzed for 25(OH) vitamin D, and the level of insufficiency was defined as < 50 nmol/L. A clinical oral examination was performed applying commonly used indices in epidemiological studies of dental caries, dental erosion, enamel defects, gingival bleeding, and oral hygiene. Serum vitamin D was used as exposure variable in multivariable regression analyses to estimate the associations between insufficient vitamin D level, JIA disease status, and oral conditions, with adjustments for age, sex, geographical region, BMI, seasonal blood sampling, and parental education.

**Results:**

Among the 223 participants with JIA, 97.3% were Caucasians, 59.2% were girls, and median age was 12.6 years. Median disease duration was 4.6 years, and 44.4% had oligoarticular JIA. Mean serum vitamin D level was 61.4 nmol/L and 29.6% had insufficient levels. Vitamin D levels did not differ between sexes, but between regions, iso-BMI categories, age groups, and seasons for blood sampling. Insufficient vitamin D levels were associated with dentin caries (adjusted OR 2.89, 95% CI 1.43–5.86) and gingival bleeding (adjusted OR 2.36, 95% CI 1.10–5.01). No associations were found with active JIA disease or more severe disease characteristics.

**Conclusion:**

In our study, nearly 30% had vitamin D insufficiency, with a particularly high prevalence among adolescents. Vitamin D insufficiency was associated with dentin caries and gingival bleeding, but not with JIA disease activity. These results point to the need for a multidisciplinary approach in the follow-up of children with JIA, including an increased focus on vitamin D status and oral health.

**Supplementary Information:**

The online version contains supplementary material available at 10.1186/s12903-022-02349-1.

## Background

Juvenile idiopathic arthritis (JIA) is the most common chronic rheumatic condition in children and adolescents worldwide, affecting more girls than boys. There are geographical differences in prevalence and incidence, and the distribution of JIA categories, medical treatment options, and levels of disease activity and severity differ [1, 2]. The disease may cause severe joint damage, impaired skeletal growth, functional disability, uveitis, and pain [3]. Arthritis of the temporomandibular joint (TMJ) is common, and it may lead to oral and dentofacial deformities such as abnormal skeletal growth of the jaws with facial asymmetry or mandibular retrognathia, and malocclusion [4, 5]. Common orofacial symptoms are temporomandibular dysfunction, with painful and restricted jaw movements due to myalgia or arthralgia [5, 6].

Poorer oral hygiene, more dental caries, and gingival inflammation have been found in JIA compared to controls, possibly due to JIA-related disability and pain [7, 8]. New treatment strategies including biologic disease-modifying anti-rheumatic drugs (DMARDs) appear to have reduced some of the detrimental effects of the disease [9]. Some of the more recent studies have not found differences in the prevalence of dental caries in JIA compared to controls [10–12], but more dental plaque and gingival inflammation are still observed [12, 13].

Vitamin D is synthesized in the skin after exposure to natural sunlight or absorbed through the dietary and supplemental intake. The vitamin D status depends on several factors such as pigmentation of the skin, amount of sun exposure, the latitude of living, season, vitamin D intake, age, sex, overweight/obesity, malabsorption, and medication such as corticosteroids [14]. Receptors for vitamin D are expressed in many types of tissues and cells, indicating a potential influence on several biological processes, such as modification of the adaptive and innate immune system, with anti-microbial and anti-inflammatory effects, and suppression of autoimmune responses [15, 16]. Inadequate vitamin D levels have been associated with several extra-skeletal, and autoimmune diseases [17, 18] and chronic pain conditions [19]. Vitamin D plays a crucial role in preserving phosphate and calcium homeostasis and enables normal mineralization, growth, and bone remodeling. Long-lasting vitamin D deficiency may lead to rickets, muscle weakness, bone pain, and growth impairment in children [20]. Vitamin D deficiency has also been linked to enamel defects and increased risk of dental caries [21]. The development and mineralization of both the baby’s primary teeth during pregnancy and permanent teeth after birth are vulnerable to disturbances in vitamin D and mineral metabolism [21]. This is supported by a recent mother-baby pair study where maternal vitamin D levels (< 50 nmol/L) during the third trimester of pregnancy, were associated with higher caries experience in the primary dentition of children at 6 years of age compared to children of mothers with sufficient third-trimester vitamin D levels [22]. Vitamin D may also influence oral health conditions through its anti-inflammatory and anti-microbial effects [23].

Studies of serum vitamin D status in children and adolescents with JIA are often characterized by small sample sizes and a lack of healthy controls and show inconsistent results regarding serum vitamin D levels, the prevalence of insufficiency and deficiency, and the association between serum vitamin D status and disease activity [24–30]. However, inadequate vitamin D levels appear to be common among young individuals with JIA [31]. Serum vitamin D levels, JIA activity, and oral health conditions might be affected by ethnicity, lifestyle, dietary habits, socio-economic and environmental factors, and also genetics [3, 14, 32, 33].

To our knowledge, no studies have looked at associations between insufficient vitamin D levels and oral conditions in JIA. We aimed to investigate vitamin D status in a cohort of Norwegian children and adolescents with JIA and study the associations between vitamin D insufficiency and JIA disease characteristics, and vitamin D insufficiency and oral conditions in this group.

## Methods

This is a cross-sectional study based on baseline data from the Norwegian Juvenile Idiopathic Arthritis study (The NorJIA Study, https://norjia.com/), a longitudinal multicenter study taking place from 2015 to 2020. Eligible children with JIA from three geographically spread regions in Norway (latitude 60.4°–69.6° N) were recruited from out-patient Pediatric Rheumatology clinics at the Departments of Pediatrics, Haukeland University Hospital in Bergen (western Norway), St. Olavs University Hospital in Trondheim (central Norway), and University Hospital of North Norway in Tromsø (northern Norway). These clinics were responsible for all children and adolescents with pediatric rheumatologic conditions in their regions. Inclusion and baseline visits took place over two years. Inclusion criteria for the NorJIA study were a diagnosis of JIA according to the ILAR classification criteria [34], age 4–16 years, and parental/patient consent. There were no exclusion criteria. In this study, additional inclusion criteria were participation in both the medical and oral examination, and available serum from the study visit for vitamin D analyses.

### Data collection

Data collection followed a standardized protocol, including a medical examination performed by pediatricians with experience in pediatric rheumatology, an oral examination performed by experienced dentists and dental specialists, parent/patient-reported questionnaires, imaging, and laboratory tests. The medical assessment included registration of demographic data, age at disease onset, disease duration, JIA category, disease status, past and present medication, and clinical examination with active joint count. The participant’s weight in kilograms (kg) and standing height in centimeters (cm) were measured. Body mass index (BMI) was calculated using the formula: BMI = (weight [kg]/(height [meters(m)])^2^) with adjustments for age and gender, giving iso-BMI groups corresponding to the following adult BMI groups; underweight: < 18.5, normal weight: 18.5–24.9, overweight: 25–29.9, obesity: ≥ 30) according to The International Obesity Task Force recommendations [35, 36]. The parents filled in questionnaires about self-reported dietary and supplemental vitamin D intake on behalf of children < 12 years of age, and older children filled in the questionnaires by themselves.

Information about parental education was reported by the parents or patients if old enough to come alone to the study visit. The four education levels: (1) primary school with 10 years of education, (2) high school with additional 2 or 3 years of education, (3) ≤ 4 years of university education, and 4) ≥ 5 years of university education, were dichotomized into low (level 1 and 2) and high education level (level 3 and 4). Since we did not have data on socioeconomic determinants other than parental education level, this was considered a proxy variable.

After the medical examinations, an oral clinical examination including bitewing radiographs was performed at either the Oral Health Centre of Expertise in western Norway (TkV), The Center for Oral Health Services and Research (TkMidt) in central Norway, or the Public Dental Health Service Competence Centre of Northern Norway (TkNN).

### Measures

#### Disease status and pain

Disease status was categorized into three groups; remission off medication, inactive disease, and active disease, according to Wallace and the American College of Rheumatology (ACR) provisional criteria [37, 38]. Inactive disease (on or off medication) included no active arthritis, no splenomegaly, generalized lymphadenopathy, serositis, fever, or rash as a result of JIA, no active uveitis, normal levels of erythrocyte sedimentation rate (ESR), and C-reactive protein (CRP), no morning stiffness exceeding 15 min, and physician’s global assessment of disease activity (MDgloVAS) on a 21-numbered visual analog scale (0 = no active disease, 10 = maximum active disease) indicating no active disease. Clinical remission off medication was defined as twelve months of maintained inactive disease and no anti-rheumatic medication [37]. Active disease included continuous active disease or flare [38]. Self-reported disease-related pain was reported by the patient if nine years or older, otherwise by the parents, on a 21-numbered circle VAS pain (0 = no pain, 10 = maximum pain) [39]. The following JIA-related characteristics associated with disease activity and/or poor disease outcome were considered as outcome variables in the regression analyses; individuals who at the study visit (a) were not in remission off medication, (b) had one or more active joints, (c) had a disease duration of ≥ 4 years, (d) were classified into other categories than oligoarticular persistent JIA, (e) had previously used or were using disease-modifying anti-rheumatic drugs (DMARDs) at the visit, or (f) reported disease-related pain (VAS pain > 0).

#### Medication

Previous and ongoing medication was registered and divided into the following treatment groups; DMARDs or no DMARDs, and systemic corticosteroids or no systemic corticosteroids. DMARDs included the following synthetic DMARDs; methotrexate, hydroxychloroquine, cyclosporine, or mycophenolate mofetil, and the following biologic DMARDs; etanercept, infliximab, adalimumab, tocilizumab, abatacept, certolizumab, golimumab, or rituximab. Systemic corticosteroids included oral or intravenous corticosteroids. Likewise, previous, and ongoing non-steroidal anti-inflammatory drugs (NSAIDs) and intraarticular injections were registered. A dichotomized variable was constructed distinguishing between children with ongoing and/or previous medication with DMARDs (DMARDs ever) and children who had never taken any DMARDs. The variable was used to adjust for high JIA disease burden in the regression analyses.

#### Oral health assessment

All participants underwent oral examinations performed by experienced dentists or dental specialists who before and during the study period underwent theoretical and practical training, and calibration exercises (Additional file 1). The oral health assessment included a thorough oral examination, clinical photos, and bitewing radiographs from the age of five if there was interproximal molar contact. The structured oral examination consisted of standard epidemiological indices of dental caries (including caries lesions confined in enamel; grades 1–2, and in dentin; grades 3–5. See Additional file 2: Table S1 for additional information), dental erosion, enamel defects, gingival bleeding, and oral hygiene. As the extensive oral examinations were performed in children and adolescents with various tooth shedding and eruption stages, only index teeth were assessed, and simplified diagnostic tools were used in order not to exhaust the participants. We did not include assessment for oral hygiene and gingival bleeding for participants under 10 years of age as it would have been a too high burden for them.

#### Dental caries

Designated surfaces of four index teeth in each participant were assessed by clinical examination and evaluation of bitewing radiographs, and graded for caries according to a detailed five-graded diagnostic tool [40]. Individual caries status was determined, and a dichotomized variable was constructed, distinguishing between participants with ≥ 1 surface of dentin caries or without dentin caries including either primary or permanent molars depending on the age of the participant. We did not include enamel caries in our study. When referring to our study results, the term dentin caries is used. See Additional file 2: Table S1, for additional information.

#### Dental erosion

Designated surfaces of six index teeth in children 4–5 years, and four teeth in participants ≥ 10 years of age were assessed for erosive wear by using two 4-graded diagnostic tools [41, 42]. The group of 6–9-year-old children with mixed dentition (both primary and permanent teeth) were excluded. Individual status of erosion on lingual/palatal surfaces and concavities on the occlusal cusps (cuppings) was determined by combining the two scores. A dichotomized variable was constructed distinguishing between participants with ≥ 1 surface with erosion /cupping or without erosion /cupping, including either primary or permanent molars depending on the age of the participant. See Additional file 2: Table S2, and S3, for additional information.

#### Enamel defects

Mineralization defects of tooth enamel were evaluated by using a modified version of the basic Enamel Defects Index (EDI) [43, 44]. Enamel defects were registered at designated surfaces of four primary index teeth in those < 10 years, and eight permanent index teeth in participants ≥ 10 years of age. Individual status including the type of enamel defect and the number of affected surfaces was determined, and dichotomized variables were constructed for each type of enamel defect (hypoplasia, opacity, and post-eruptive breakdown), distinguishing between participants with ≥ 1 surface with enamel defects or without enamel defects, including either primary or permanent molars depending on the age of the participant. See Additional file 2: Table S4, for additional information.

#### Gingival bleeding

A modified version of the Gingival Bleeding Index (GBI) [45] was used to measure bleeding after gentle probing of the gingival sulcus at designated surfaces of six permanent index teeth in participants ≥ 10 years of age, using a periodontal probe with 0.5 mm ball-ended tip. Individual GBI was calculated as the sum of bleeding points, divided by the total number of points examined for each participant. The GBI scores were divided into three levels. This was done by finding the lowest and the highest scores registered, and dividing the highest score by three, giving the lowest third, the middle third, and the highest third of scores A dichotomized variable was constructed to distinguish between individuals with middle/high levels and those with low levels of gingival bleeding. See Additional file 2 for additional information.

#### Oral hygiene

An evaluation of oral hygiene was done in participants ≥ 10 years of age, by assessing the amount of debris (dental plaque) and dental calculus at designated surfaces of six permanent index teeth, using a modified version of the simplified Oral Hygiene Index (OHI-S) [46]. The number of surfaces with debris and calculus, respectively, were divided by the total number of surfaces examined, giving individual simplified Debris index (DI-S) and Calculus index (CI-S) scores. The OHI-S consists of the sum of the DI-S and CI-S. The DI-S and OHI-S scores registered were divided into three levels. This was done by finding the lowest and the highest DI-S and OHI-S scores of the total cohort, respectively, and dividing the highest score by three, giving the lowest third, the middle third, and the highest third of scores. Dichotomized variables were constructed, to distinguish between participants with middle/high and low DI-S and OHI-S, respectively. See Additional file 2: Table S5, for additional information. The dichotomized oral health variables were used as outcome variables in the multivariable regression analyses.

### Vitamin D analyses

One blood sample per individual was drawn in conjunction with the medical examinations at the three study sites. All samples were frozen at − 80 °C, sent to Trondheim, and analyzed for 25(OH) vitamin D at the Department of Medical Biochemistry, St. Olavs Hospital, using ultra-performance liquid chromatography-tandem mass spectrometry (Acquity UPLC^©^ I Class with Xevo TQ-S MSMS by Waters). Serum vitamin D2 and D3 were measured with a validated method following the Norwegian Accreditation standard ISO 151189. Precision and accuracy were systematically followed to withhold the demands for a coefficient of variance set to 5.7% for vitamin D2 and 6.0% for D3. The method was monitored by participation in The Vitamin D External Assessment Scheme (DEQAS) proficiency testing program. DEQAS has close links to the US National Institute for Standards and Technology (NIST) and the Vitamin D Standardization Program (VDSP). Defined vitamin D levels were < 30 nmol/L = deficient, 30–49 nmol/L = insufficient, 50–74 nmol/L = sufficient, and ≥ 75–150 nmol/L = desired level. In our study, we chose to use the recommended level for insufficiency of < 50 nmol/L according to the Nordic Nutrition Recommendation 2012, which is in line with guidelines from the National Academy of Medicine (the former Institute of Medicine (IOM)) [47]. A dichotomized variable was constructed, distinguishing between insufficient < 50 nmol/L and sufficient ≥ 50 nmol/L serum levels, and used as an exposure variable in the regression analyses. To look for seasonal differences, the samples drawn in winter (Dec-Feb), spring (Mar-May), summer (Jun-Aug), and fall (Sept-Nov) were compared. A variable was constructed for seasonal blood sampling and was adjusted for in the regression analyses. In the text, 25(OH) vitamin D is referred to as vitamin D and serum vitamin D.

### Vitamin D intake

Estimates of vitamin D intake were based on an extensive self-reported food frequency questionnaire (FFQ). The FFQ was completed by the parents/caregivers of children < 12 years, while those ≥ 12 years of age answered the questionnaires themselves. The mean daily intake (µg/day) of vitamin D from food and vitamin D supplements was estimated, respectively, and the total mean daily intake of vitamin D was derived by adding the two together. Total mean daily intake was categorized into < 10 and ≥ 10 µg/day, which is the recommended daily intake according to The Norwegian Directorate of Health and Nordic Nutrition Recommendations 2012 (Nordic Nutrition Recommendations 2012; diva-portal.org) and presented as frequencies and percentages. The nutritional estimates were performed by an experienced clinical nutritionist (IL). Additional information is found in Additional file 3.

### Statistics

The normal distribution of continuous variables was assessed by studying Q-Q plots. Descriptive statistics of serum, dietary and supplemental vitamin D, demographics, seasons, anthropometrics, and JIA disease characteristics are presented. Furthermore, descriptive statistics of serum vitamin D and vitamin D intake are presented in relation to oral health conditions. Continuous variables are presented with means and standard deviations (SD) or medians and interquartile range (IQR). Categorical variables are presented as frequencies and percentages. Multivariable logistic regression analyses were performed to assess the association of exposure to insufficient levels of serum vitamin D (at a cut-off point of 50 nmol/L) in relation to several JIA-related outcomes chosen to reflect disease activity/severity, and also in relation to oral health outcomes. Only adjusted models are presented. Model 1 was adjusted for potential confounders identified by a priori knowledge [48] including age, sex, geographical region, seasons for blood sampling, and iso-BMI. For oral health-related outcomes, Model 1 was further adjusted for previous/ongoing treatment with DMARDs in Model 2. Further, we also conducted analyses for JIA-related as well as oral health-related outcomes, with further adjustments for parental education. The odds ratios (OR) and their corresponding 95% confidence intervals (CI) are presented. Statistical significance was assumed when *p* < 0.05.

Given 61 participants with dentin caries and 157 without dentin caries, the power was calculated to be 91% to detect a difference corresponding to 0.5 SD, and 75% to detect a difference of 0.4 SD in the mean value of a continuous variable between the two groups. For dichotomous variables, the power was calculated close to 79% to detect a difference in proportions between the groups corresponding to 0.5 in participants with dentin caries and 0.3 in participants without dentin caries. (Alpha = 0.05, 2-sided test).

Statistical analyses were performed using IBM SPSS Statistics version 26.

## Results

Of the 360 eligible children and adolescents with JIA, 228 were included in the NorJIA study (response rate of 63.3%), as previously described [11]. Four of the 228 did not participate in the oral examination and were therefore excluded from this study. In addition, one child had four index teeth extracted, making it impossible to perform the oral examinations according to the procedure, and this child was therefore also excluded. The remaining 223 had both medical and oral data recorded and blood samples for analysis of 25(OH) vitamin D and were eligible for this study. Among these 223 individuals, 97.3% were Caucasians, 59.2% were girls, the median age was 12.6 years (IQR 9.4–14.7), and 23.8% were either overweight or obese (Table 1). The median age at disease onset was 5.8 years (IQR 2.3–10.3). Oligoarticular disease (persistent or extended) was found in 44.4%, and 87.0% were not in remission off medication. Only 1.8% were on corticosteroids, while 66.4% had ongoing medication with synthetic or biologic DMARDs. At the study visit, 22.9% had active joints, and among the 136 (62.8%) individuals reporting disease-related pain (VAS pain > 0), the mean VAS pain score was 2.0 (± 2.2).

**Table 1:** Clinical characteristics of children in the NorJIA study population, N = 223

Characteristics at study visit	Values
Girls, n (%)	132 (59.2)
Age at examination, median years, (IQR)	12.6 (9.4–14.7)
Caucasian, n (%)	217 (97.3)
Overweight/obesity^a^, n (%)	53 (23.8)
Parental education level^b^, n (%)	
Low	64 (30.3)
High	147 (69.7)
Age at disease onset, median years, (IQR)	5.8 (2.3–10.3)
Disease duration, median years, (IQR)	4.6 (2.6–8.2)
Oligoarticular arthritis, persistent or extended, n (%)	99 (44.4)
Not in remission off medication^c^, n (%)	194 (87.0)
Ongoing medication^d^, n (%)	
DMARDs	148 (66.4)
bDMARDs	87 (39.0)
Systemic corticosteroids	4 (1.8)
NSAIDs	71 (31.8)
Number of children with active joints, n (%)	51 (22.9)
VAS pain > 0^ e^, mean (± SD)	2.0 (± 2.2)

NorJIA, Norwegian juvenile idiopathic arthritis; IQR, inter-quartile range (25th–75th percentile); DMARDs, disease-modifying anti-rheumatic drugs, including both synthetic and biologic DMARDs; bDMARDs, only biologic DMARDs; NSAIDs, non-steroid anti-inflammatory drugs; VAS, Visual Analogue Scale; SD, standard deviation

^a^Age- and sex-adjusted body mass index (iso-BMI) according to the International Obesity Task Force recommendations corresponding to adult BMI (overweight: BMI = 25–29.9, obesity BMI ≥ 30), overweight and obesity combined

^b^Low level was defined as primary and high school (education ≤ 13 years), High was defined as university education, the parent with the highest education level defined the level for both parents, n = 211 (12 missing)

^c^Disease status categorized according to Wallace et al. 2004/2011

^d^Medication ongoing at study visit: *DMARDs* include both synthetic (methotrexate, hydroxychlorochine, cyclosporine or mycophenolate mofetil) and/or biologic (etanercept, infliximab, adalimumab, tocilizumab, abatacept, certolizumab, golimumab or rituximab), *Systemic corticosteroids* oral or intravenous

^e^Mean self-reported disease-related pain measured on a 21-numbered circle VAS scale, 0–10 cm (0 = no pain, 10 = maximum pain, 5 missing), VAS > 0 n = 136

### Mean serum vitamin D levels and daily vitamin D intake

The mean serum vitamin D level was 61.4 nmol/L (SD ± 22.3), and 29.6% had insufficient levels, including 6.7% with deficient levels below 30 nmol/L (Table 2). Seasonal differences were found ranging from the lowest mean vitamin D level of 51.4 (SD ± 21.2) measured in winter to the highest of 72.5 (SD ± 21.6) nmol/L in summer. The estimated mean daily intake of vitamin D from food and supplements was 11.2 µg/day (SD ± 12.2), and 66.1% of the participants had an intake, including supplements, lower than the recommended 10 µg/day. Supplemental vitamin D was taken by 75.2%, and intake decreased with increasing age. Participants from the northern part of Norway had the poorest vitamin D intake from food and supplements (Table 2). There was a weak positive albeit statistically significant correlation between the mean daily intake of vitamin D from food/supplements and serum vitamin D level; Pearson correlation r = 0.186, *p* < 0.006, indicating that children with lower vitamin D intake had lower serum vitamin D levels.

**Table 2:** Serum and dietary vitamin D status in the NorJIA study population, N = 223

Serum and dietary vitamin D		Values
Serum 25(OH) vit. D^a^, nmol/L, mean (± SD)		61.4 (± 22.3)
Serum 25(OH) vit. D in categories, n (%)		
Deficient < 30 (nmol/L)		15 (6.7)
Insufficient 30–49 (nmol/L)		51 (22.9)
Sufficient 50–74 (nmol/L)		100 (44.8)
Desired ≥ 75–150 (nmol/L)		57 (25.6)
Seasonal serum 25(OH) vit. D^b^, nmol/L, mean (± SD)		
Winter, December-February (n = 49)		51.4 (± 21.2)
Spring, March–May (n = 66)		55.6 (± 18.8)
Summer, June–August (n = 25)		72.5 (± 21.6)
Fall, September–November (n = 83)		68.8 (± 22.1)
Daily vit. D intake^c^, µg/day, mean (± SD)		11.2 (± 12.2)
Northern Norway (n = 51)		7.7 (± 8.4)
Western Norway (n = 74)		9.9 (± 11.8)
Central Norway (n = 93)		14.0 (± 13.6)
Inadequate intake^d^, < 10 µg/day, n (%)		144 (66.1)
Supplemental vit. D intake, n (%)		164 (75.2)
Northern Norway		34 (66.7)
Western Norway		49 (66.2)
Central Norway		81 (87.1)

NorJIA study, Norwegian juvenile idiopathic arthritis study; vit. D, vitamin D; SD, standard deviation

^a^Serum 25(OH) vitamin D collected during the study visits taking place throughout the calendar year, except for July

^b^Serum 25(OH) vitamin D collected during the four specified seasons

^c^Estimates of daily vit. D intake (dietary and supplemental) based on data from self-reported food frequency questionnaires (FFQ), n = 218 (5 participants did not fill in the FFQ), 164 participants reported supplemental vitamin D intake

^d^10 µg/day is the daily recommended intake of vit. D according to The Norwegian Directorate of Health and Nordic Nutrition Recommendations 2012 (5 participants did not fill in the FFQ)

Regional differences in mean serum vitamin D levels were observed, with the lowest levels seen in participants from northern Norway, 56.1 nmol/L (SD ± 26.0), and the highest, 66.0 nmol/L (SD ± 21.3) in central Norway (Table 3). The vitamin D levels in boys and girls did not differ, but levels decreased with increasing age; from 69.2 nmol/L (SD ± 20.3) in the youngest age group (4–9 years) to 56.1 nmol/L (SD ± 22.1) in adolescents (13–16 years). Likewise, the proportion of individuals with insufficient vitamin D levels increased with age. The vitamin D levels varied according to iso-BMI from 67.2 nmol/L (SD ± 23.0) in underweight, 64.0 nmol/L (SD ± 21.6) in normal weight, to 39.5 nmol/L (SD ± 13.1) in obese individuals (Table 3). Mean serum vitamin D levels also varied between different JIA categories. However, the number of subjects in some categories was small. In groups with adequate sample size, participants with oligoarticular persistent JIA had the highest mean vitamin D levels, 63.6 nmol/L (SD ± 21.7) versus 57.7 nmol/L (SD ± 20.6) in the polyarticular rheumatoid factor negative group. We found no clear differences in serum vitamin D levels according to disease status or medication (Table 3). JIA-related pain was reported by 62.8%, but we found no significant difference in serum vitamin D levels between participants reporting pain and those who did not.

**Table 3:** Serum 25(OH) vitamin D levels according to demographics, anthropometrics, and disease characteristics

Characteristics	Total N	Serum 25(OH) vit. D nmol/L mean (± SD)	Serum 25(OH) vit. D < 50 nmol/L n (%)
Geographical regions			
North Norway	56	56.1 (± 26.0)	26 (46.4)
West Norway	74	59.9 (± 19.5)	20 (27.0)
Central Norway	93	66.0 (± 21.3)	20 (21.5)
Sex			
Girls	132	61.7 (± 22.5)	39 (29.5)
Boys	91	61.2 (± 22.1)	27 (29.7)
Age groups, years			
4–9	62	69.2 (± 20.3)	8 (12.9)
10–12	58	62.7 (± 22.5)	17 (29.3)
13–16	103	56.1 (± 22.1)	41 (39.8)
Iso-BMI^a^			
Underweight	13	67.2 (± 23.0)	3 (23.1)
Normal weight	157	64.0 (± 21.6)	39 (24.8)
Overweight	43	55.6 (± 22.9)	17 (39.5)
Obesity	10	39.5 (± 13.1)	7 (70.0)
Disease duration			
< 4 years	91	62.1 (± 23.8)	26 (28.6)
≥ 4 years	132	61.1 (± 21.3)	40 (30.3)
JIA categories^b^			
Systemic	7	80.7 (± 26.6)	1 (14.3)
Oligoarticular persistent	77	63.6 (± 21.7)	18 (23.4)
Oligoarticular extended	22	59.0 (± 19.8)	7 (31.8)
Polyarticular RF negative	50	57.7 (± 20.6)	20 (40.0)
Polyarticular RF positive	4	70.8 (± 39.9)	1 (25.0)
Psoriatic arthritis	9	53.1 (± 21.0)	5 (55.6)
Enthesitis-related arthritis	23	56.7 (± 20.0)	7 (30.4)
Undifferentiated arthritis	31	64.5 (± 25.0)	7 (22.6)
Disease status^c^			
Remission off medication	29	58.0 (± 22.8)	10 (34.5)
Inactive disease	105	63.3 (± 23.3)	30 (28.6)
Active disease	89	60.5 (± 21.0)	26 (29.2)
Active joints			
No	172	61.3 (± 22.0)	50 (29.1)
Yes	51	62.1 (± 23.5)	16 (31.4)
Medication			
DMARDs^d^			
Never used	52	58.3 (± 21.2)	15 (28.8)
Ever used	171	62.4 (± 22.6)	51 (29.8)
Ongoing	148	62.8 (± 22.9)	44 (29.7)
Systemic corticosteroids^e^			
Never used	175	61.6 (± 22.6)	48 (27.4)
Ever used	48	60.9 (± 21.5)	18 (37.5)
Ongoing	4	77.8 (± 42.6)	1 (25.0)
Pain^f^			
VAS pain = 0	82	59.1 (± 20.8)	26 (31.7)
VAS pain > 0	136	62.9 (± 22.4)	37 (27.2)

DMARDs, disease-modifying anti-rheumatic drugs; vit. D, vitamin D; SD, standard deviation; ILAR, International League of Association for Rheumatology; RF, rheumatoid factor; VAS, Visual Analogue Scale

^a^Body mass index (BMI) adjusted for age and sex according to the International Obesity Task Force recommendations corresponding to adult BMI (kg/m2) (underweight: < 18.5, normal weight: 18.5–24.9, overweight: 25–29.9, obesity: ≥ 30)

^b^According to the ILAR classification criteria

^c^Disease status was categorized according to the definition by Wallace et al. 2004/2011. Remission off medication = inactive disease off medication for ≥ 12 months. Inactive = inactive disease on medication for < 6 months, or off medication < 12 months, or remission on medication (inactive disease on medication for ≥ 6 months). Active = continuous active disease or flare

^d^Medication; never used, ever used (= previous, and ongoing at study visit)

*DMARDs* include both synthetic (methotrexate, hydroxychlorochine, cyclosporine or mycophenolate mofetil) and biologic (etanercept, infliximab, adalimumab, tocilizumab, abatacept, certolizumab, golimumab or rituximab)

^e^*Systemic corticosteroids* include oral or intravenous corticosteroids

^f^Self-reported disease-related pain measured on a 21-numbered circle VAS scale (0 = no pain, 10 = maximum pain) (5 missing)

### Oral health and vitamin D

Children and adolescents with dentin caries, 61/218 (27.9%), had lower mean serum vitamin D levels than those without dentin caries; 52.0 (SD ± 23.4) versus 65.2 (SD ± 21.1) nmol/L, respectively (Table 4). Five participants did not have a registration of caries. The highest caries prevalence was found in the group of 13–16-year-old participants with insufficient serum vitamin D; 25/41 (58.5%) compared to 4/16 (20.0%) and 2/8 (20.0%) in the age groups 10–12 and 4–9 years with insufficient vitamin D levels, respectively (results not shown). Participants with middle/high levels of gingival bleeding had lower vitamin D levels compared to those with low bleeding levels, and a similar, but less pronounced pattern was seen for enamel defects. Three participants did not have a registration of gingival bleeding. The opposite trend was seen for the simplified Debris Index, and simplified Oral Hygiene Index, while no difference in mean vitamin D levels was found between participants with or without dental erosion. Two participants did not have the examination for erosion.

**Table 4:** Serum 25(OH) vitamin D levels according to oral health conditions

Oral health conditions	Total N	Serum 25(OH) vit. D, nmol/LMean (± SD)
Caries^a^		
No	157	65.2 (± 21.1)
Yes	61	52.0 (± 23.4)
Hypoplasia^b^		
No	213	61.7 (± 22.4)
Yes	10	55.6 (± 20.2)
Opacity^b^		
No	132	62.5 (± 20.8)
Yes	91	60.0 (± 24.3)
Post-eruptive breakdown^b^		
No	213	61.8 (± 22.6)
Yes	10	55.4 (± 14.0)
Dental erosion^c^		
No	74	59.3 (± 24.4)
Yes	97	59.5 (± 21.1)
GBI^d^		
Low	114	59.5 (± 20.4)
Middle/high	44	55.4 (± 27.4)
DI-S^e^		
Low	87	55.2 (± 22.6)
Middle/high	54	60.7 (± 22.9)
OHI-S^f^		
Low	84	55.2 (± 22.4)
Middle/high	57	60.5 (± 23.2)

SD, standard deviation; vit., vitamin; GBI, Gingival Bleeding Index; DI-S, Debris Index simplified (dental bacterial plaque); OHI-S, Oral Hygiene Index simplified

^a^Caries included dentin caries (grade 3–5, Amarante 1998) and filled teeth in primary second molars and permanent first molars (5 did not have caries registration)

^b^Enamel defects (Brook et al. 2001, Elcock et al. 2006) include Hypoplasia = areas on the tooth with thin enamel, Opacity = opaque white or yellow hypomineralized areas, Post-eruptive breakdown = loss of enamel after tooth eruption

^c^Dental erosion (Hasselkvist et al. 2010, Johansson et al. 1996) include buccal and lingual surface erosion, and occlusal cupping. Children aged 4–5 and 10–16 years included (2 did not have the examination)

^d^GBI (Ainamo & Bay, 1975), categorized into two levels; the lowest third and a combination of the middle and highest thirds of GBI scores. Children aged 10–16 years included (3 did not have the examination)

^e^DI-S (Greene & Vermillion, 1964), categorized into two levels; the lowest third and a combination of the middle and highest thirds of DI-S scores. Children aged 10–16 years included (20 missing as those with fixed orthodontic appliances were excluded in these analysis)

^f^OHI-S (Greene & Vermillion, 1964) = the sum of Debris Index and Calculus index, categorized into two levels; the lowest third and a combination of the middle and highest thirds of OHI-S scores. Children aged 10–16 years included (20 missing due to fixed orthodontic appliances)

### Associations between vitamin D status, and JIA-related or oral health outcomes

Multivariable regression analyses suggested no significant association between insufficient serum vitamin D levels and several JIA-related outcome variables chosen to reflect disease activity, pain, and/or poor disease outcome (Table 5, Model 1). However, significant inverse associations were found between serum vitamin D and dentin caries (OR 2.89 (95% CI 1.43–5.86) and gingival bleeding (OR, 2.36, 95% CI, 1.10–5.01) in the adjusted model 2 (Table 6). No significant associations were observed between serum vitamin D and enamel defects (including hypoplasia, opacity, post-eruptive breakdown), dental erosion, simplified debris index, or simplified oral hygiene index. After further adjustment for parental education, the odds ratios slightly changed for JIA-, and oral health-related outcomes (Additional file 4: Table S6).

**Table 5:** Multivariable logistic regression analysis between serum vitamin D levels and JIA- related outcomes

JIA-related outcomes	Yes/No N	Serum 25(OH) vit. Das exposure	Model 1 Adjusted^a^ OR (95% CI)
Disease duration ≥ 4 years^b^	92/65	≥ 50 nmol/L	1 (ref.)
	40/26	< 50 nmol/L	0.83 (0.43–1.60)
Not oligo persistent JIA^c^	98/59	≥ 50 nmol/L	1 (ref.)
	48/18	< 50 nmol/L	1.54 (0.77–3.10)
DMARDs ever used^d^	120/37	≥ 50 nmol/L	1 (ref.)
	51/15	< 50 nmol/L	1.29 (0.58–2.87)
Not in remission off medication^e^	138/19	≥ 50 nmol/L	1 (ref.)
	56/10	< 50 nmol/L	0.81 (0.32–2.07)
Active joints^f^	35/122	≥ 50 nmol/L	1 (ref.)
	16/50	< 50 nmol/L	1.07 (0.51–2.24)
VAS pain > 0^ g^	99/56	≥ 50 nmol/L	1 (ref.)
	37/26	< 50 nmol/L	0.64 (0.33–1.24)

The column Yes/No, N shows the number of participants with (Yes) and without (No) the JIA-related outcome within each of the two vitamin D exposure groups ≥ 50 nmol/L and < 50 nmol/L

Vit., vitamin; JIA, juvenile idiopathic arthritis; OR, odds ratio; CI, confidence interval; DMARDs, disease-modifying anti-rheumatic drugs; VAS, Visual Analog Scale; iso-BMI, Body Mass Index adjusted for age and sex, corresponding to adult BMI according to International Obesity Task Force; ILAR, International League of Association for Rheumatology

^a^Model 1: Adjusted for age, sex, geographical region, iso-BMI, and blood sampling performed in 4 seasons (summer, fall, winter, spring)

^b^Disease duration was categorized into: < 4 years and ≥ 4 years

^c^JIA categories defined according to the ILAR classification criteria and categorized into oligoarticular persistent JIA (the mildest form), and all other JIA categories

^d^DMARDs include both synthetic (methotrexate, hydroxychloroquine, cyclosporine, mycophenolate mofetil) and biologic (etanercept, infliximab, adalimumab, tocilizumab, abatacept, certolizumab, golimumab, rituximab) and categorized into never used, and ever used (= previous, or ongoing medication)

^e^Disease activity (Wallace et al. 2004/2011), categorized into not in remission off medication, and remission off medication

^f^Active joints at the study visit = children without active joints, and those with one or more active joints

^g^Self-reported disease-related pain measured on a 21-numbered circle VAS scale (0 = no pain, 10 = maximum pain) and categorized into no pain (VAS = 0), and pain (VAS > 0) (5 missing)

**Table 6:** Multivariable logistic regression analysis between serum vitamin D levels and oral health

Oral health outcomes	Yes/no N	Serum 25(OH) vit. D as exposure	Model 1^a^ adjusted OR (95% CI)	Model 2^b^ djusted OR (95% CI)
Caries^c^	30/123	≥ 50 nmol/L	1 (ref.)	1 (ref.)
	31/34	< 50 nmol/ L	2.88 (1.44–5.78)	2.89 (1.43–5.86)
Hypoplasia^d^	6/151	≥ 50 nmol/L	1 (ref.)	1 (ref.)
	4/62	< 50 nmol/L	2.01 (0.48–8.46)	2.04 (0.49–8.52)
Opacity^d^	63/94	≥ 50 nmol/L	1 (ref.)	1 (ref.)
	28/38	< 50 nmol/L	1.03 (0.49–1.75)	0.93 (0.49–1.76)
Post-eruptive breakdown^d^	8/149	≥ 50 nmol/L	1 (ref.)	1 (ref.)
	2/64	< 50 nmol/L	0.50 (0.91–2.79)	0.47 (0.08–2.66)
Dental erosion^e^	65/48	≥ 50 nmol/L	1 (ref.)	1 (ref.)
	32/26	< 50 nmol/L	1.13 (0.54–2.34)	1.13 (0.54–2.34)
GBI, middle/high^f^	21/79	≥ 50 nmol/L	1 (ref.)	1 (ref.)
	23/35	< 50 nmol/L	2.32 (1.09–4.93)	2.36 (1.10–5.01)
DI-S, middle/high^g^	38/49	≥ 50 nmol/L	1 (ref.)	1 (ref.)
	16/38	< 50 nmol/L	0.72 (0.33–1.61)	0.72 (0.32–1.60)
OHI-S, middle/high^h^	39/48	≥ 50 nmol/L	1 (ref.)	1 (ref.)
	18/36	< 50 nmol/L	0.82 (0.37–1.82)	0.81 (0.36–1.79)

In the column Yes/No, N shows the number of participants with (Yes) and without (No) the JIA-related outcome within each of the two vitamin D exposure groups ≥ 50 nmol/L and < 50 nmol/L

OR, odds ratio; CI, confidence interval; vit. D, vitamin D; iso-BMI, Body Mass Index adjusted for age and sex; DMARDs, disease-modifying anti-rheumatic drugs; GBI, Gingival Bleeding Index; DI-S, simplified Debris Index; OHI-S, = simplified Oral Hygiene Index

^a^Model 1: Adjusted for age, sex, geographical region, iso-BMI, and season for blood sampling (summer, fall, winter, spring)

^b^Model 2: Model 1, and adjusted for DMARDs ever/never used

^c^Caries included dentin caries (grades 3–5) and filled teeth. Dichotomized into no caries (no) and caries (yes) (5 did not have caries registration)

^d^Enamel defects: Hypoplasia, Opacity and Post-eruptive breakdown (Brook et al. 2001, Elcock et al. 2006), categorized into not present(no), and present (yes). Children 4–16 years included

^e^Dental erosion (Hasselkvist et al. 2010, Johansson et al. 1996) categorized into not present (no), and present (yes). Children aged 4–5 and 10–16 years included (2 did not have the examination)

^f^Modified GBI (Ainamo & Bay, 1975), dichotomized into two levels of bleeding: Low = the lowest third (no), and Middle/High = a combination of the middle and highest third of scores (yes). Children aged 10–16 years included (3 missing)

^g^DI-S (Greene & Vermillion, 1964), dichotomized into two levels: Low = the lowest third (no), and Middle/High = a combination of the middle and highest third of scores (yes). Children aged 10–16 years included (20 missing due to fixed orthodontic appliances)

^h^Modified OHI-S (Greene & Vermillion, 1964), dichotomized into two levels: low = the lowest third (no), and middle/high = a combination of the middle and highest third of scores (yes). Children aged 10–16 years included (20 missing due to fixed orthodontic appliances)

## Discussion

In this multicenter cross-sectional study of 4–16-year-old children with JIA, nearly one-third had insufficient serum vitamin D levels. Consistent with other studies [25–30, 49], we found no difference between boys and girls, but in contrast, our results showed clear differences in vitamin D related to age. We did not find any associations between serum vitamin D levels and JIA-related disease characteristics, but there was an association between vitamin D insufficiency and dentin caries, and gingival bleeding.

The relatively few studies that compare vitamin D levels in JIA to controls have shown inconsistent results. Some studies have found no difference in vitamin D levels [29, 30], while others have found lower [26, 28] or higher mean vitamin D levels in JIA *versus* controls [25, 49]. The lack of sun exposure during a large part of the year in Norway has led to strong national health recommendations for vitamin D supplementation for all children and adults in the form of cod-liver oil or other vitamin D supplements. This may be one explanation for the higher mean vitamin D results in our Norwegian JIA cohort, compared to two studies performed in Turkey [27, 28]. The geographical differences in serum vitamin D levels in JIA are further reflected by three studies performed in central and south America, and Canada where the vitamin D levels in JIA were considerably higher than in our study population [24, 30, 49]. This might be the result of more exposure to sunlight, more frequent vitamin D fortification of foods and beverages, or a higher intake of supplements.

Studies of the association between serum vitamin D and JIA disease activity show divergent results. Some have found an association between vitamin D deficiency and increased disease activity [25–27], while others, consistent with our results, have not [24, 28, 30]. Significant differences in vitamin D levels between JIA categories, with the lowest levels seen in polyarticular and systemic JIA, have been reported [26]. This is in line with our results, demonstrating a trend towards lower vitamin D levels in the oligoarticular extended and polyarticular RF negative group.

Comparisons between studies are hampered by several factors such as different study designs, different measures used to indicate JIA disease activity and oral health conditions, geographically and demographically disparate study populations, different cut-off values for vitamin D insufficiency and deficiency, the use of varying methods for measuring serum vitamin D, and often a lack of information about dietary and supplemental vitamin D intake.

We did not find significantly more JIA-related pain or more severe JIA disease characteristics among participants with insufficient vitamin D levels compared to those with sufficient levels in our study cohort, where more than 66% were on synthetic or biologic DMARDs. Children with fewer treatment opportunities and thus higher JIA-related disability and pain might have lower serum vitamin D levels, and poorer oral health.

The assessment of serum 25(OH) vitamin D is considered the most accurate to indicate a person’s vitamin D status, as it gives an indication of the amount of vitamin D obtained through dietary intake and sun exposure [50]. Compared to its’ active form (1,25-dihydroxy vitamin D), 25(OH) vitamin D also has higher concentrations and a longer half-life in serum, which has led to the presumption that serum vitamin D levels could remain fairly unchanged over a short time. A Norwegian study showed small changes in 25(OH) vitamin D concentrations in adults over a longer time (14 years) [51]. Seventy-five percent of the individuals with serum vitamin D levels below 30 nmol/L at baseline still had levels below 50 nmol/L by the end of follow-up, which gives some support to the clinical use of a single measurement for vitamin D in adults. However, the latter study only includes an adult population and does not assess the association with oral health conditions.

A recent systematic review investigating the association between low prenatal, or childhood vitamin D levels and dental caries, found that there is evidence supporting an association between serum vitamin D levels below 75 nmol/L and caries experience in children [52]. They also concluded that low prenatal or childhood vitamin D levels should be considered potential risk factors for caries in children. However, there is a shortage of confirming evidence from high-quality studies. Most of the studies concerning vitamin D and caries in children have used a cross-sectional design and their analyses are based on single one-time measurements of serum vitamin D. This may be because of the ethical aspects of multiple blood sampling in healthy children. A single measure of vitamin D may not fully explain a long-standing and multi-factorial condition such as dental caries or determine caries risk in children.

High-quality studies of the association between vitamin D and dental caries in JIA are lacking. Therefore, further investigation in a prospective longitudinal study setting is warranted.

Incidence and prevalence of JIA are high in Norway, and we have shown that insufficient serum vitamin D levels are prevalent in these children, particularly in adolescents. Even though we did not find any association between vitamin D and disease activity, the associations between vitamin D insufficiency and dentin caries, and gingival bleeding may have implications for JIA disease outcome. With respect to the immunosuppressed state of many patients with JIA on immunomodulatory treatment, oral infections followed by bacteremia and risk for systemic infection might pose an additional threat. Maintaining good oral health is beneficial both for teeth and oral mucosa, but also for general health and well-being. Increased focus on individual vitamin D status in the follow-up of children and adolescents with JIA, recognition of low vitamin D intake, limited sun exposure, and the need for supplements in most children, particularly adolescents, are important messages to pediatric rheumatologists.

### Strengths and limitations

The strengths of this study include the sample size, the wide age range from 4 to 16 years, and a representative study group including all the JIA categories. The participants were recruited from three regions in Norway (latitude 60.4°–69.6° N) and the examinations and serum vitamin D measurements were performed throughout the calendar year. Data collection and clinical examinations were performed by experienced pediatricians and dentists, following a standardized protocol with validated JIA disease activity measurements, and standardized pre-calibrated oral examination techniques. The vitamin D measurements were performed in the same laboratory, using validated and standardized methods. Furthermore, we adjusted for an extensive range of potential confounders in the regression analyses.

Our study also has some limitations. Some JIA categories had too few participants to allow meaningful grouping for sub-analyses. Also, as this was a cross-sectional study, only associations between serum vitamin D levels, JIA characteristics, and oral health-related outcomes could be assessed, not causality. Although we adjusted for many confounders in the regression analyses, there are still potential confounders that we have not adjusted for, such as JIA-related disability, and JIA-related pain which could potentially affect dietary intake, serum vitamin D levels, and oral health. We did not consider JIA-related disability in this study. We found no association between vitamin D insufficiency and JIA-related pain. Medication with DMARDs and JIA-related pain were two of six outcome variables chosen to serve as a proxy for more severe JIA. As JIA-related pain may be related to DMARDs, we chose not to adjust for JIA-related pain in addition to DMARDs in our regression analyses of vitamin D and oral health outcomes. Only one measurement for serum 25(OH) vitamin D per child was made. This remains a limitation with respect to determining its association with dental caries, given that caries is a multi-factorial and dynamic condition that develops over time. Another limitation is the absence of a control group for the serum vitamin D analyses and oral health outcomes. Information about dietary intake and estimates of vitamin D intake was based on a self-reported food frequency questionnaire (FFQ) which may be affected by recall and information bias. The modified diagnostic tools used in this study may complicate comparisons with other studies.

## Conclusion

In our study of Norwegian children with JIA, nearly one-third had vitamin D insufficiency. The highest prevalence was seen among adolescents. We did not find an association between vitamin D insufficiency and JIA disease activity, but there was evidence of associations between insufficient vitamin D levels and dentin caries, and gingival bleeding. The association between vitamin D and oral health conditions in JIA should be further investigated, preferentially in a prospective longitudinal study setting. Our results point to the need for a multidisciplinary approach in the follow-up of children and adolescents with JIA, increased awareness of vitamin D insufficiency, and more focus on oral health in JIA.

## Supplementary Information

**Additional file 1: Supplemental file S1.** Calibration.

**Additional file 2: Supplemental file S2.** Oral health assessments.

**Additional file 3: Supplemental file S3.** Dietary and supplemental vitamin D intake.

**Additional file 4: Supplemental file S4.** Additional adjustments in the regression analyses.

## Data Availability

The dataset used in this study can be made available at reasonable request to the corresponding author.

## Abbreviations

JIA: Juvenile idiopathic arthritis
DMARDs: Disease-modifying antirheumatic drugs
SD: Standard deviation
CI: Confidence interval
IQR: Inter-quartile range
Iso-BMI: Body mass index in children adjusted for age and sex, corresponding to adult BMI groups
VAS: Visual analog scale
ILAR: International League of Associations for Rheumatology
RF: Rheumatoid factor
EDI: Enamel Defects Index
SEPRS: Simplified erosion partial recording system
GBI: Gingival Bleeding Index
DI: Debris Index (Plaque Index)
CI: Calculus Index
OHI-S: Oral Hygiene Index—simplified

## References

[1] Consolaro A, Giancane G, Alongi A, van Dijkhuizen EHP, Aggarwal A, Al-Mayouf SM (2019). Phenotypic variability and disparities in treatment and outcomes of childhood arthritis throughout the world: an observational cohort study. Lancet Child Adolesc Health.

[2] Thierry S, Fautrel B, Lemelle I, Guillemin F (2014). Prevalence and incidence of juvenile idiopathic arthritis: a systematic review. Joint Bone Spine.

[3] Ravelli A, Martini A (2007). Juvenile idiopathic arthritis. Lancet.

[4] Abramowicz S, Levy JM, Prahalad S, Travers CD, Angeles-Han ST (2019). Temporomandibular joint involvement in children with juvenile idiopathic arthritis: a preliminary report. Oral Surg Oral Med Oral Pathol Oral Radiol.

[5] Stoustrup P, Glerup M, Bilgrau AE, Küseler A, Verna C, Christensen AE (2020). Cumulative incidence of orofacial manifestations in early juvenile idiopathic arthritis: a regional, three-year cohort study. Arthritis Care Res (Hoboken).

[6] Fischer J, Skeie MS, Rosendahl K, Tylleskär K, Lie S, Shi XQ (2020). Prevalence of temporomandibular disorder in children and adolescents with juvenile idiopathic arthritis—a Norwegian cross-sectional multicentre study. BMC Oral Health.

[7] Welbury RR, Thomason JM, Fitzgerald JL, Steen IN, Marshall NJ, Foster HE (2003). Increased prevalence of dental caries and poor oral hygiene in juvenile idiopathic arthritis. Rheumatology (Oxford).

[8] Leksell E, Ernberg M, Magnusson B, Hedenberg-Magnusson B (2008). Intraoral condition in children with juvenile idiopathic arthritis compared to controls. Int J Paediatr Dent.

[9] Giancane G, Muratore V, Marzetti V, Quilis N, Benavente BS, Bagnasco F (2019). Disease activity and damage in juvenile idiopathic arthritis: methotrexate era versus biologic era. Arthritis Res Ther.

[10] Feres de Melo AR, Ferreira de Souza A, de Oliveira Perestrelo B, Leite MF. Clinical oral and salivary parameters of children with juvenile idiopathic arthritis. Oral Surg Oral Med Oral Pathol Oral Radiol. 2014;117(1):75–80. 10.1016/j.oooo.2013.08.024 24332330

[11] Gil EG, Åstrøm AN, Lie SA, Rygg M, Fischer J, Rosén A (2021). Dental caries in children and adolescents with juvenile idiopathic arthritis and controls: a multilevel analysis. BMC Oral Health.

[12] Skeie MS, Gil EG, Cetrelli L, Rosén A, Fischer J, Åstrøm AN (2019). Oral health in children and adolescents with juvenile idiopathic arthritis—a systematic review and meta-analysis. BMC Oral Health.

[13] Frid P, Baraniya D, Halbig J, Rypdal V, Songstad NT, Rosèn A (2020). Salivary oral microbiome of children with juvenile idiopathic arthritis: a Norwegian cross-sectional study. Front Cell Infect Microbiol.

[14] Holick MF (2007). Vitamin D deficiency. N Engl J Med.

[15] Wang Y, Zhu J, DeLuca HF (2012). Where is the vitamin D receptor?. Arch Biochem Biophys.

[16] Bishop E, Ismailova A, Dimeloe SK, Hewison M, White JH. Vitamin D and immune regulation: antibacterial, antiviral, anti-inflammatory. JBMR Plus. 2020;5(1). 10.1002/jbm4.10405 PMC7461279 32904944

[17] Antonucci R (2018). Vitamin D deficiency in childhood: old lessons and current challenges. J Pediatric Endocrinol Metab.

[18] Bouillon R, Marcocci C, Carmeliet G, Bikle D, White JH, Dawson-Hughes B (2019). Skeletal and extraskeletal actions of vitamin D: current evidence and outstanding questions. Endocr Rev.

[19] Shipton EA, Shipton EE (2015). Vitamin D and pain: Vitamin D and Its role in the aetiology and maintenance of chronic pain states and associated comorbidities. Pain Res Treat.

[20] Carpenter TO, Shaw NJ, Portale AA, Ward LM, Abrams SA, Pettifor JM (2017). Rickets. Nat Rev Dis Primers.

[21] Foster BL, Hujoel P. P. Vitamin D in dentoalveolar and oral health. In: Feldman D, editor. Vitamin D (Fourth Edition). 1: biochemistry, physiology and diagnostics. Fourth ed. Science Direct: Academic Press; 2018. p. 497–519.

[22] Beckett DM, Broadbent JM, Loch C, Mahoney EK, Drummond BK, Wheeler BJ (2022). Dental consequences of Vitamin D deficiency during pregnancy and early infancy—an observational study. Int J Environ Res Public Health..

[23] Uwitonze AM, Murererehe J, Ineza MC, Harelimana EI, Nsabimana U, Uwambaye P (2018). Effects of vitamin D status on oral health. J Steroid Biochem Mol Biol.

[24] Pelajo CF, Lopez-Benitez JM, Kent DM, Price LL, Miller LC, Dawson-Hughes B (2012). 25-hydroxyvitamin D levels and juvenile idiopathic arthritis: is there an association with disease activity?. Rheumatol Int.

[25] Sengler C, Zink J, Klotsche J, Niewerth M, Liedmann I, Horneff G (2018). Vitamin D deficiency is associated with higher disease activity and the risk for uveitis in juvenile idiopathic arthritis—data from a German inception cohort. Arthritis Res Ther.

[26] Stagi S, Bertini F, Cavalli L, Matucci-Cerinic M, Brandi ML, Falcini F (2014). Determinants of vitamin D levels in children, adolescents, and young adults with juvenile idiopathic arthritis. J Rheumatol.

[27] Çomak E, Doğan ÇS, Uslu-Gökçeoğlu A, Akbaş H, Özdem S, Koyun M (2014). Association between vitamin D deficiency and disease activity in juvenile idiopathic arthritis. Turk J Pediatr.

[28] Dağdeviren-Çakır A, Arvas A, Barut K, Gür E, Kasapçopur Ö (2016). Serum vitamin D levels during activation and remission periods of patients with juvenile idiopathic arthritis and familial Mediterranean fever. Turk J Pediatr.

[29] Lien G, Selvaag AM, Flatø B, Haugen M, Vinje O, Sørskaar D (2005). A two-year prospective controlled study of bone mass and bone turnover in children with early juvenile idiopathic arthritis. Arthritis Rheum.

[30] Munekata RV, Terreri MT, Peracchi OA, Len C, Lazaretti-Castro M, Sarni RO (2013). Serum 25-hydroxyvitamin D and biochemical markers of bone metabolism in patients with juvenile idiopathic arthritis. Braz J Med Biol Res.

[31] Finch SL, Rosenberg AM, Vatanparast H (2018). Vitamin D and juvenile idiopathic arthritis. Pediatr Rheumatol Online J.

[32] Gazzaz AZ, Carpiano RM, Aleksejuniene J (2021). Socioeconomic status, social support, and oral health-risk behaviors in Canadian adolescents. J Public Health Dent.

[33] Schroth RJ, Levi JA, Sellers EA, Friel J, Kliewer E, Moffatt ME (2013). Vitamin D status of children with severe early childhood caries: a case-control study. BMC Pediatr.

[34] Petty RE, Southwood TR, Manners P, Baum J, Glass DN, Goldenburg J (2004). International League of Associations for Rheumatology classification of juvenile idiopathic arthritis: second revision, Edmonton, 2001. J Rheumatol..

[35] Cole TJ, Lobstein T (2012). Extended international (IOTF) body mass index cut-offs for thinness, overweight and obesity. Pediatr Obes.

[36] Júlíusson PB, Hjelmesæth J, Bjerknes R, Roelants M. New curves for body mass index among children and adolescents. Tidsskr Nor Laegeforen. 2017;137(18). 10.4045/tidsskr.17.0570 28972329

[37] Wallace CA, Giannini EH, Huang B, Itert L, Ruperto N (2011). American College of Rheumatology provisional criteria for defining clinical inactive disease in select categories of juvenile idiopathic arthritis. Arthritis Care Res (Hoboken).

[38] Wallace CA, Ruperto N, Giannini E (2004). Preliminary criteria for clinical remission for select categories of juvenile idiopathic arthritis. J Rheumatol.

[39] Filocamo G, Davì S, Pistorio A, Bertamino M, Ruperto N, Lattanzi B (2010). Evaluation of 21-numbered circle and 10-centimeter horizontal line visual analog scales for physician and parent subjective ratings in juvenile idiopathic arthritis. J Rheumatol.

[40] Amarante E, Raadal M, Espelid I (1998). Impact of diagnostic criteria on the prevalence of dental caries in Norwegian children aged 5, 12 and 18 years. Community Dent Oral Epidemiol.

[41] Hasselkvist A, Johansson A, Johansson AK (2010). Dental erosion and soft drink consumption in Swedish children and adolescents and the development of a simplified erosion partial recording system. Swed Dent J.

[42] Johansson AK, Johansson A, Birkhed D, Omar R, Baghdadi S, Carlsson GE (1996). Dental erosion, soft-drink intake, and oral health in young Saudi men, and the development of a system for assessing erosive anterior tooth wear. Acta Odontol Scand.

[43] Brook AH, Hallonsten AL, Poulsen S, Anreasen J, Koch G, Yeung CA, Dosanjh T. The development of a new index to measure enamel defects. In: Brook AH, editor. Dental morphology shefield. Academic Press. 2001. p. 59–66.

[44] Elcock C, Lath DL, Luty JD, Gallagher MG, Abdellatif A, Bäckman B, et al. The new Enamel Defects Index: testing and expansion. Eur J Oral Sci. 2006;114 Suppl 1:35–8; discussion 9–41, 379. 10.1111/j.1600-0722.2006.00294.x 16674660

[45] Ainamo J, Bay I (1975). Problems and proposals for recording gingivitis and plaque. Int Dent J.

[46] Greene JC, Vermillion JR (1964). The simplified oral hygiene index. J Am Dent Assoc.

[47] Bouillon R (2017). Comparative analysis of nutritional guidelines for vitamin D. Nat Rev Endocrinol.

[48] Hernán MA, Hernández-Díaz S, Werler MM, Mitchell AA (2002). Causal knowledge as a prerequisite for confounding evaluation: an application to birth defects epidemiology. Am J Epidemiol.

[49] Finch SL, Rosenberg AM, Kusalik AJ, Maleki F, Rezaei E, Baxter-Jones A (2021). Higher concentrations of vitamin D in Canadian children with juvenile idiopathic arthritis compared to healthy controls are associated with more frequent use of vitamin D supplements and season of birth. Nutr Res.

[50] Zerwekh JE (2008). Blood biomarkers of vitamin D status. Am J Clin Nutr.

[51] Jorde R, Sneve M, Hutchinson M, Emaus N, Figenschau Y, Grimnes G (2010). Tracking of serum 25-hydroxyvitamin D levels during 14 years in a population-based study and during 12 months in an intervention study. Am J Epidemiol.

[52] Carvalho Silva C, Mendes R, Manso MDC, Gavinha S, Melo P (2020). Prenatal or childhood serum levels of vitamin D and dental caries in paediatric patients: a systematic review. Oral Health Prev Dent.

