# Supplementary material for: Vitamin D, oral health, and disease characteristics in juvenile idiopathic arthritis: a multicenter cross-sectional study
Source: BMC Oral Health. 2022 Aug 8;22:333. doi: 10.1186/s12903-022-02349-1 (PMC9361556; doi:10.1186/s12903-022-02349-1)
Supplement: Supplementary file 2 — Additional file 2: Supplemental file S2. Oral health assessments. [file 12903_2022_2349_MOESM2_ESM.docx]

**Supplemental File S2**. Oral health assessments

The oral examination took place in a dental office under standard dental lighting using a plain mouth mirror, clinical probe, and a 0.5 mm ball-ended periodontal probe.

**Dental caries**

Dental caries data was registered on five surfaces (buccal, mesial, distal, palatal/lingual, and occlusal) as decayed or filled surfaces in four primary molars (55, 65, 75, 85) in children < 10 years, and four permanent first molars (16, 26, 36, 46) in children ≥ 10 years of age. Bitewing radiographs were taken from five years of age, on the condition that intermolar contact was established. No bitewings were taken when the child had fixed orthodontic appliances, and in this case, only the occlusal surfaces of the teeth were assessed for caries. A 5-graded diagnostic tool was used (39); grade 1-2 indicated caries lesions confined to enamel only, and grade 3-5 indicated caries involving dentin **(**See table s1). Teeth extracted due to caries were too few and the missing component was not included in the analyses.

**Table s1.** Diagnostic criteria for dental caries (simplified from (39))

| Grade | Criteria |
| --- | --- |
| 1 | White or brown discolorations in enamel, without cavitation. Dental radiograph shows no visible signs of caries on occlusal surfaces, but approximal lesions can be seen as radiolucencies in the outer 1/2 of enamel |
| 2 | Small cavity formed in the enamel surface or discoloration in occlusal fissures with surrounding grey/opaque enamel. Dental radiograph shows radiolucency in the inner 1/2 of the enamel |
| 3 | Moderate size cavity in enamel with exposed dentin, verified by probing. Dental radiograph shows radiolucency in the outer 1/3 of dentin |
| 4 | Large cavity formation in enamel and moderate cavity in dentin. Dental radiograph shows radiolucency in the middle 1/3 of dentin |
| 5 | Considerably large cavity formation. Dental radiograph shows radiolucency in the inner 1/3 of dentin |

**Dental erosion**

Two 4-graded diagnostic tools were used to describe dental erosive wear; Johansson et al. (41) for dental erosion on lingual/palatal surfaces of maxillary anterior teeth (Table s2) and Hasselkvist et al. (40) for cupping on occlusal surfaces of permanent first molars and primary molars (Table s3). According to the Simplified Erosion Partial Recording System (SEPRS), described by Hasselkvist et al. (40), the palatal surfaces of two incisors were assessed (in children 4-5 years: 51 and 61, in children ≥10 years of age: 11 and 21). The assessment was modified as the buccal surfaces were not included. Additionally, the occlusal surfaces were assessed in four primary molars (55, 65, 75, 85) in children 4-5 years, and in two permanent first molars (36 and 46) in children ≥ 10 years of age.

**Table s2.** Ordinal scale for grading severity of dental erosion (simplified from (41))

| Grade | Criteria |
| --- | --- |
| 0 | No visible changes in tooth anatomy, enamel surface intact |
| 1 | Smoothened enamel, surface structures have partially or completely disappeared. Enamel surface is shiny, matt irregular, “melted”, rounded or flat, Minor changes in tooth anatomy |
| 2 | Enamel surface as in grade 1. Tooth anatomy clearly changed, faceting or concavity formation (“cupping”) within the enamel, without exposed dentin |
| 3 | Enamel surface as in grades 1 and 2. Tooth anatomy severely changed (close to dentin-exposure of large surfaces) or dentin exposed by ≤ 1/3 |
| 4 | Enamel surface as in grades 1, 2, and 3. Dentin exposed by ≥ 1/3, or pulp visible through the dentin |

**Table s3.** Ordinal scale for grading of “cuppings” (40)

| Grade | Criteria |
| --- | --- |
| 0 | No cupping/intact anatomy of cusp tip |
| 1 | Rounded cusp tip* |
| 2 | Cupping ≤ 1 mm |
| 3 | Cupping > 1 mm |
| 4 | Cuppings merged: At least two cuppings merged on the same tooth |

*Changed morphology compared to the assumed original anatomy of the tooth at eruption

Individual erosion values for each participant were calculated by adding the erosion and cupping scores together.

**Enamel defects**

Mineralization defects of tooth enamel were evaluated using an index by Elcook et al (43), derived from the basic Enamel Defects Index (EDI) according to Brook et al (42) (Table s4). In participants ≥ 10 years, enamel defects were registered on the surfaces of eight index teeth: The buccal surfaces of central incisors (11, 21, 31, 41) and the occlusal, and palatal/lingual surfaces of permanent first molars (16, 26, 36, 46). In children < 10 years, four primary molars were assessed: (55, 65, 75, 85). Three types of enamel defects were described.

**Table s4**. Category definitions of the basic version of the Enamel Defects Index (EDI) (42)

| Defect | Definition |
| --- | --- |
| Hypoplasia | A quantitative enamel defect associated with reduced enamel thickness; enamel may be translucent or opaque |
| Opacity | A qualitative enamel defect associated with alterations in the translucency of enamel; enamel has normal thickness with a smooth surface, but the defective area may be white, yellow, or brown, with a demarcated or diffuse border |
| Post-eruptive breakdown | Loss of surface enamel after tooth eruption |

**Gingival bleeding**

Using a modified version of the Gingival Bleeding Index (GBI) according to Ainamo & Bay (44), gingival bleeding after gentle vertical probing in the upper part of the gingival sulcus, without the original horizontal movement along the tooth surface (hence modified version), was registered. Gingival bleeding was recorded as present or not present at three measuring points (mesial, medial, and distal) for each buccal and palatal surface of six permanent index teeth; three teeth in the upper jaw (16, 26, 11), and buccal and lingual surfaces of three teeth in the lower jaw (36, 46, 31). Individual GBI was calculated as the sum of bleeding points, divided by the total number of points examined for each child.

**Oral Hygiene**

Bacterial plaque (debris) and dental calculus were registered on the buccal surfaces of permanent incisors (11, 31), the permanent first molars (16, 26), and the lingual surfaces of the permanent first molars (36, 46). A score from 0-3 for each buccal and lingual surface was estimated according to Greene & Vermillion (45) (Table s5). The simplified Debris Index (DI-S) and Calculus Index (CI-S) were both calculated by dividing the sum of buccal and lingual scores by the total number of examined surfaces. Individual oral hygiene index (OHI-S) scores were calculated by adding DI-S and CI-S together. The registration did not include sub-gingival calculus (hence the modified version). Teeth not fully erupted or teeth with fixed orthodontic appliances were excluded.

**Table s5.** Scoring of the simplified OHI-S index (45)

| Scale | Criteria |
| --- | --- |
| 0 | No detectable debris or calculus |
| 1 | Debris or calculus covering not more than 1/3^rd^ of the examined tooth surface |
| 2 | Debris or calculus covering more than 1/3^rd^ but not more than 2/3^rds^ of the examined tooth surface |
| 3 | Debris or calculus covering more than 2/3^rds^ of the examined tooth surface |

**References**

39. Amarante E, Raadal M, Espelid I. Impact of diagnostic criteria on the prevalence of dental caries in Norwegian children aged 5, 12 and 18 years. Community Dent Oral Epidemiol. 1998;26(2):87-94.

40. Hasselkvist A, Johansson A, Johansson AK. Dental erosion and soft drink consumption in Swedish children and adolescents and the development of a simplified erosion partial recording system. Swed Dent J. 2010;34(4):187-95.

41. Johansson AK, Johansson A, Birkhed D, Omar R, Baghdadi S, Carlsson GE. Dental erosion, soft-drink intake, and oral health in young Saudi men, and the development of a system for assessing erosive anterior tooth wear. Acta Odontol Scand. 1996;54(6):369-78.

42. Brook AH EC, Hallonsten AL,Poulsen S,Anreasen J,Koch G,Yeung CA,Dosanjh T. The development of a new index to measure enamel defects. In: Brook AH, ed Dental Morphology Shefield: Academic Press. 2001(2001):59-66.

43. Elcock C, Lath DL, Luty JD, Gallagher MG, Abdellatif A, Bäckman B, et al. The new Enamel Defects Index: testing and expansion. Eur J Oral Sci. 2006;114 Suppl 1:35-8; discussion 9-41, 379.

44. Ainamo J, Bay I. Problems and proposals for recording gingivitis and plaque. Int Dent J. 1975;25(4):229-35.

45. Greene JC, Vermillion JR. THE SIMPLIFIED ORAL HYGIENE INDEX. J Am Dent Assoc. 1964;68:7-13.
