# Supplementary material for: Vitamin D, oral health, and disease characteristics in juvenile idiopathic arthritis: a multicenter cross-sectional study
Source: BMC Oral Health. 2022 Aug 8;22:333. doi: 10.1186/s12903-022-02349-1 (PMC9361556; doi:10.1186/s12903-022-02349-1)
Supplement: Supplementary file 3 — Additional file 3: Supplemental file S3. Dietary and supplemental vitamin D intake. [file 12903_2022_2349_MOESM3_ESM.docx]

**Supplemental File S3** - Dietary and supplemental vitamin D intake

Estimations of vitamin D intake were based on an extensive food frequency questionnaire (FFQ). The nutritional content was calculated using the Norwegian food composition table*. In addition, nutrient values not found in the food composition tables were retrieved from websites of vitamin D supplement producers, dairies, and pharmacies. For vitamin D, 18 questions from the FFQ were included. Food items were cow’s milk, vitamin D-fortified milk, vegan milk alternatives such as soy- and oat drinks fortified with vitamin D, margarine, butter, lean fish, fatty fish, and dietary supplements including vitamin D. The registered intake frequency of food items varied from never to several times a day, with portion sizes reported as slices, glasses, cups, and pieces. For each question, an average of the vitamin D content per 100 grams was calculated using the food composition table, multiplied by the portion size and intake frequency. For solid and liquid forms of supplements, the vitamin D content per tablet or ml was used.

The nutritional calculations were performed by an experienced clinical nutritionist (IL).

Further details on the FFQ may be provided on reasonable request. However, the questionnaire itself is in Norwegian.

*The Norwegian food composition table <https://www.kostholdsplanleggeren.no/displayfoods/?profileId=3&slotNumber=0> .
