# Supplementary material for: Vitamin D, oral health, and disease characteristics in juvenile idiopathic arthritis: a multicenter cross-sectional study
Source: BMC Oral Health. 2022 Aug 8;22:333. doi: 10.1186/s12903-022-02349-1 (PMC9361556; doi:10.1186/s12903-022-02349-1)
Supplement: Supplementary file 4 — Additional file 4: Supplemental file S4. Additional adjustments in the regression analyses. [file 12903_2022_2349_MOESM4_ESM.docx]

**Supplemental File S4.** Additional adjustments in the regression analyses

**Supplemental Table 5.** Multivariable logistic regression analysis between serum vitamin D levels and JIA- related outcomes

| **JIA-related outcomes** | **Yes/No**  **N** | **Serum 25(OH) vit. D**  **as exposure** | **Model 2**  **Adjusted^a^**  **OR (95% CI)** |
| --- | --- | --- | --- |
| **Disease duration ≥ 4 years^b^** | 92/65 | ≥ 50 nmol/L | 1 (ref.) |
|  | 40/26 | < 50 nmol/L | 0.79 (0.39-1.59) |
| **Not oligo persistent JIA^c^** | 98/59 | ≥ 50 nmol/L | 1 (ref.) |
|  | 48/18 | < 50 nmol/L | 1.42 (0.69-2.93) |
| **DMARDs ever used^d^** | 120/37 | ≥ 50 nmol/L | 1 (ref.) |
|  | 51/15 | < 50 nmol/L | 1.10 (0.48-2.52) |
| **Not in remission off medication^e^** | 138/19 | ≥ 50 nmol/L | 1 (ref.) |
|  | 56/10 | < 50 nmol/L | 0.73 (0.28-1.92) |
| **Active joints^f^** | 35/122 | ≥ 50 nmol/L | 1 (ref.) |
|  | 16/50 | < 50 nmol/L | 1.00 (0.46-2.21) |
| **VAS pain > 0^g^** | 99/56 | ≥ 50 nmol/L | 1 (ref.) |
|  | 37/26 | < 50 nmol/L | 0.56 (0.28-1.14) |

The column Yes/No, N shows the number of participants with (Yes) and without (No) the JIA-related outcome within each of the two vitamin D exposure groups ≥ 50 nmol/L and < 50 nmol/L. Vit. = vitamin, JIA = juvenile idiopathic arthritis, OR = odds ratio, CI = confidence interval, DMARDs = disease-modifying anti-rheumatic drugs, VAS = visual analog scale, iso-BMI = body mass index adjusted for age and sex, corresponding to adult BMI according to International Obesity Task Force, ILAR = International League of Association for Rheumatology.

^a^ Model 2: Model 1 (adjusted for age, sex, geographical region, iso-BMI, and season for blood sampling (summer, fall, winter, spring)) and parental education level serving as a proxy for socioeconomic status

^b^ Disease duration was categorized into: < 4 years and ≥ 4 years

^c^ JIA categories are defined according to the ILAR classification criteria and categorized into oligoarticular persistent JIA (the mildest form), and all other JIA categories

^d^ DMARDs include both synthetic (methotrexate, hydroxychloroquine, cyclosporine, mycophenolate mofetil) and biologic (etanercept, infliximab, adalimumab, tocilizumab, abatacept, certolizumab, golimumab, rituximab) and categorized into never used, and ever used (= previous or ongoing medication)

^e^ Disease activity (Wallace et al 2004/2011), categorized into not in remission off medication, and remission off medication

^f^ Active joints at the study visit = children without active joints, and those with one or more active joints

^g^ Self-reported disease-related pain measured on a 21-numbered circle VAS scale (0 = no pain, 10 = maximum pain) and categorized into no pain (VAS = 0), and pain (VAS > 0) (5 missing)

**Supplemental Table 6**. Multivariable logistic regression analysis between serum vitamin D

levels and oral health

| **Oral health outcomes** | **Yes/No**  **N** | **Serum**  **25(OH) vit. D**  **as exposure** | **Model 3^a^**  **Adjusted**  **OR (95% CI)** |
| --- | --- | --- | --- |
| **Caries^c^** | 30/123 | ≥ 50 nmol/L | 1 (ref.) |
|  | 31/34 | < 50 nmol/ L | 2.91 (1.37-6.22) |
| **Hypoplasia^d^** | 6/151 | ≥ 50 nmol/L | 1 (ref.) |
|  | 4/62 | < 50 nmol/L | 2.89 (0.65-12.78) |
| **Opacity^d^** | 63/94 | ≥ 50 nmol/L | 1 (ref.) |
|  | 28/38 | < 50 nmol/L | 1.06 (0.54-2.09) |
| **Post-eruptive breakdown^d^** | 8/149 | ≥ 50 nmol/L | 1 (ref.) |
|  | 2/64 | < 50 nmol/L | 0.66 (0.11-3.77) |
| **Dental erosion^e^** | 65/48 | ≥ 50 nmol/L | 1 (ref.) |
|  | 32/26 | < 50 nmol/L | 1.54 (0.69-3.43) |
| **GBI, Middle/High^f^** | 21/79 | ≥ 50 nmol/L | 1 (ref.) |
|  | 23/35 | < 50 nmol/L | 2.13 (0.95-4.76) |
| **DI-S, Middle/High^g^** | 38/49 | ≥ 50 nmol/L | 1 (ref.) |
|  | 16/38 | < 50 nmol/L | 0.63 (0.27-1.47) |
| **OHI-S, Middle/High^h^** | 39/48 | ≥ 50 nmol/L | 1 (ref.) |
|  | 18/36 | < 50 nmol/L | 0.72 (0.31-1.69) |

The column Yes/No, N shows the number of participants with (Yes) and without (No) the JIA-related outcome within each of the two vitamin D exposure groups ≥ 50 nmol/L and < 50 nmol/L. OR = odds ratio, CI = confidence interval, vit. D = vitamin D, iso-BMI = body mass index adjusted for age and sex, DMARDs = disease-modifying anti-rheumatic drugs, GBI = Gingival Bleeding Index, DI-S = simplified Debris Index, OHI-S = simplified Oral Hygiene Index.

**^a^** Model 3: Model 1&2 (adjusted for age, sex, geographical region, iso-BMI, season for blood sampling (summer, fall, winter, spring) DMARDs (previous, and ongoing, and never used) and further adjustment for parental education level that serves as a proxy for socioeconomic status

**^c^** Caries included dentin caries (grade 3-5) and filled teeth. Dichotomized into no caries (no) and caries (yes) (5 did not have caries registration)

**^d^** Enamel defects: Hypoplasia, Opacity and Post-eruptive breakdown (Brook et al 2001, Elcock et al 2006), categorized into not present(no), and present (yes). Children 4-16 years included

**^e^** Dental erosion (Hasselkvist et al 2010, Johansson et al 1996) categorized into not present (no), and present (yes). Children aged 4-5 and 10-16 years included (2 did not have the examination)

**^f^**  Modified GBI (Ainamo & Bay, 1975), dichotomized into two levels of bleeding: Low = the lowest third (no), and Middle/High = a combination of the middle and highest third of scores (yes). Children aged 10-16 years included (3 missing)

**^g^** DI-S (Greene & Vermillion, 1964), dichotomized into two levels: Low = the lowest third (no), and Middle/High = a combination of the middle and highest third of scores (yes). Children aged 10-16 years included (20 missing due to fixed orthodontic appliances)

**^h^** Modified OHI-S (Greene & Vermillion, 1964), dichotomized into two levels: Low = the lowest third (no), and Middle/High = a combination of the middle and highest third of scores (yes). Children aged 10-16 years included (20 missing due to fixed orthodontic appliances)
